# Solid-state studies and antioxidant properties of the γ-cyclodextrin·fisetin inclusion compound

**DOI:** 10.3762/bjoc.13.212

**Published:** 2017-10-13

**Authors:** Joana M Pais, Maria João Barroca, Maria Paula M Marques, Filipe A Almeida Paz, Susana S Braga

**Affiliations:** 1 QOPNA, Department of Chemistry, University of Aveiro, 3810-193 Aveiro, Portugal; 2 CICECO – Aveiro Institute of Materials, Department of Chemistry, University of Aveiro, 3810-193 Aveiro, Portugal; 3 R&D Group ‘‘Molecular Physical-Chemistry – QFM-UC”, University of Coimbra, 3004-535 Coimbra, Portugal; 4 Department of Life Sciences, University of Coimbra, 3000-456 Coimbra, Portugal

**Keywords:** antioxidant activity, co-lyophilization, fisetin, gamma-cyclodextrin, molecular encapsulation, solid-state analysis

## Abstract

Fisetin is a natural antioxidant with a wide range of nutraceutical properties, including antidiabetic, neuroprotecting, and suppression or prevention of tumors. The present work describes the preparation of a water-soluble, solid inclusion compound of fisetin with gamma-cyclodextrin (γ-CD), a cyclic oligosaccharide approved for human consumption. A detailed physicochemical analysis of the product is carried out using elemental analysis, powder X-ray diffraction (PXRD), Raman, infrared and ^13^C{^1^H} CP-MAS NMR spectroscopies, and thermal analysis (TGA) to verify fisetin inclusion and to present a hypothetical structural arrangement for the host–guest units. The antioxidant activity of the γ-CD·fisetin inclusion compound is evaluated by the DPPH assay.

## Introduction

Flavonoids, natural compounds with numerous beneficial actions on human health, including antioxidant [1], anti-inflammatory [2–3], anti-atherosclerotic [4–5], antidiabetic [6] and antitumor activities [7], are presently receiving much attention. Studies focus not only on the elucidation of their biomolecular targets but also on their applications as medicinal supplements [8] and in food fortification [9]. Examples include their content in cocoa powder [10], grains like rice, maize and wheat [11] and bread [12]. Fisetin, a flavonol, is among the most promising compounds of this class due to its broader range of activities. Besides those previously mentioned, fisetin also contributes to cancer prevention [13], helps reducing complications associated with type I diabetes [14] and features, upon oral administration, neuroprotective and memory-enhancing properties [15–16]. For this reason, fisetin is being proposed as a new approach to treat Alzheimer’s disease [17]. However, fisetin is practically insoluble in water resulting in a low oral bioavailability. This limitation can be overcome by using micro or nano-encapsulation methods that allow preparing fisetin oral liquid formulations that are well absorbed. Previously reported encapsulation strategies include liposomes [18], nanoemulsions [19], nanocochleates (which are liposome derivatives) [20], and cyclodextrin inclusion complexes [21–23]. Cyclodextrins (CDs) are naturally occurring cyclic oligosaccharides produced by bacterial degradation of starch. Native CDs have six to eight α-D-glucose units linked by α-1,4 bonds, being called α-, β- and γ-CDs, respectively [24], and are able to solubilize hydrophilic molecules such as nutraceuticals or pharmaceuticals, providing that these have the adequate size and geometry to fit into the CDs cavities [25–28]. Nowadays, native cyclodextrins are approved by the FDA and the WHO/FAO Joint Committee with the GRAS status (list of food additives that are ‘generally recognized as safe’) [9].

**Scheme 1:**
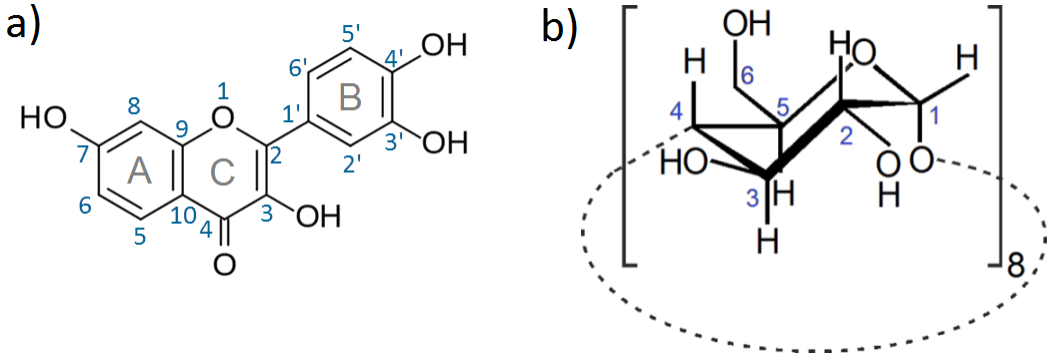
Structural representation of fisetin as guest (a) and γ-CD as host (b) used in the present study, depicting the herein adopted labeling for atoms and rings of fisetin.

The present work describes the preparation of a water-soluble, solid inclusion compound of fisetin with γ-CD (see Scheme 1 for structure and atom labeling). The formation of the γ-CD·fisetin inclusion compound as a solid product improves its stability and makes it easier to handle, transport and store. Furthermore, the used encapsulation method applied only edible ingredients: γ-CD, water, and ethanol, being thus suitable for future applications in food fortification. A detailed physicochemical analysis of the product using solid-state techniques was carried out to confirm fisetin inclusion into the cavity of γ-CD, and to present the hypothetical structural arrangement of the host–guest units. Further, the retention of the antioxidant activity in the inclusion compound was evaluated by its ability to scavenge the radical 2,2-diphenyl-1-picrylhydrazyl (DPPH).

## Results and Discussion

The present report is divided into two main parts, the first one being the preparation of the inclusion compound of fisetin with γ-CD and its characterization by a collection of solid-state techniques. The second part comprises the evaluation of the antioxidant activity of the inclusion compound by the DPPH assay, and its comparison with that of pure fisetin.

### Preparation and characterization of γ-CD·fisetin

The preparation of the γ-CD·fisetin inclusion complex was carried out by co-dissolution, followed by either co-crystallization or, when the isolation of larger amounts of a solid complex is desired, by snap freezing and subsequent freeze-drying. In all methods, the solubilization of the guest, fisetin, was achieved using ethanol as the co-solvent, as a non-toxic and environmental friendly solvent. By this way, the inclusion compound herein produced is adequate for human consumption.

The co-crystallization experiments allowed the isolation of small crystals but these were not suitable for a structural determination by single crystal X-ray diffraction. Therefore the detailed characterization of the inclusion compound was thus conducted on the freeze-dried samples. The elemental analysis data are in agreement with the values calculated for a 1:1 stoichiometry, i.e., the empirical formula C_48_H_80_O_40_·C_15_H_10_O_6_·20H_2_O. Note also the remarkable increase in the hydration status, from 7 water molecules per host molecule in γ-CD to 20 water molecules per host–guest unit in the inclusion compound.

**Vibrational spectroscopy.** FTIR spectroscopy is a quick and non-destructive method which was used for the initial assessment of the inclusion of fisetin into the γ-CD cavity. It is common to observe shifts in the bands of certain oscillators such as the carbonyl group or the vibrational modes of the aromatic ring. In the γ-CD·fisetin compound the most relevant shifts are associated with the stretching modes of the aromatic rings, as shown in Table 1 (note: for a full list of vibrational bands, see the experimental section).

**Table 1:** Selected FTIR bands for fisetin and its inclusion compound with γ-CD.^a,b^.

fisetin		γ-CD·fisetin	

calculated [29]	observed	observed	description [29]

1618	1620	1626	ν (C=C)_rings A and B_ and ν (C–O)_rings B and C_
1596	1599	1609	ν (C=C)_ring B_
1570	1572	1570	ν (C=C)_all rings_ and ν (C–O) _ring C_
1504	1510	1518	ν (C=C)_ring B_
1450	1445	1463	ν (C–O)_A-C rings bond_ and ν (C=C)_ring A_

^a^Refer to Scheme 1a for the numbering of carbon atoms and labeling of the rings of fisetin. ^b^Values given in cm^−1^.

The observation of significant blueshifts in bands associated with the catechol ring (B) is indicative of its deep penetration into the γ-CD cavity, thus confirming the formation of a true inclusion compound in the product. The band observed at 1445 cm^−1^ in pure fisetin, associated with stretching of the A and C rings, is also blueshifted to 1463 cm^−1^ in γ-CD·fisetin, thus indicating that inclusion also affects the chromenone fragment of fisetin.

**Figure 1:**
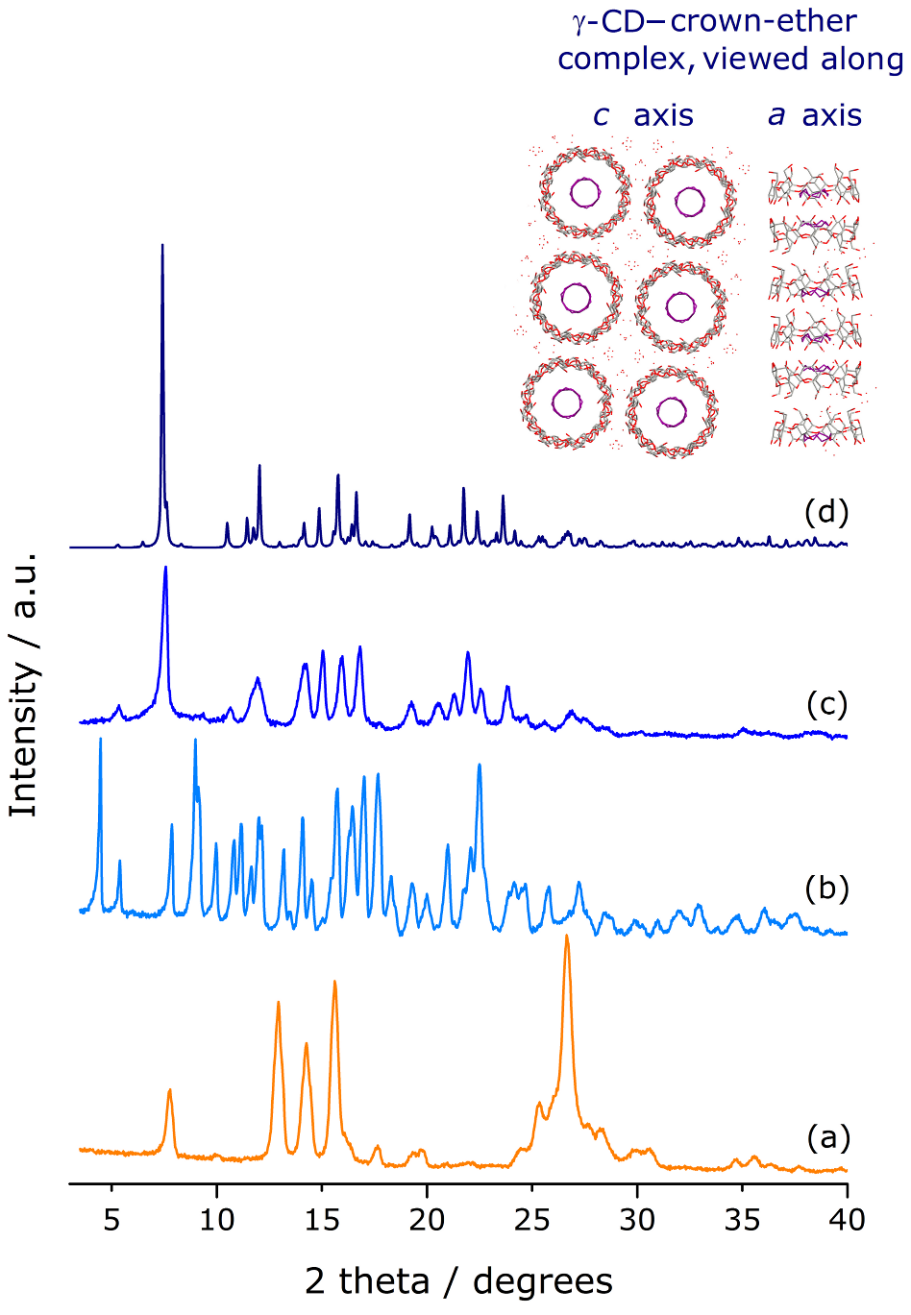
Powder X-ray diffraction patterns (PXRD) for (a) fisetin, (b) γ-CD heptahydrate and (c) the γ-CD·fisetin inclusion compound. Trace (d) was calculated from the atomic coordinates of the inclusion compound γ-CD–12-crown-4 ether [31] using the software Mercury 3.5.1 (copyright CCDC 2001–2014). The inset depicts the structure of γ-CD–12-crown-4 ether, as viewed from the top (crystallographic *c-*axis) and from the sides (crystallographic *a-*axis).

**Powder X-ray diffraction.** Powder X-ray diffraction (PXRD) of the freeze-dried product revealed it to be amorphous, an expectable result in light of the preparation method. Nonetheless, the crystallinity is easily regained by subjecting the bulk material, at ambient temperature, to a water-saturated atmosphere during ca. 16 hours (for details, see the experimental section). The PXRD of the rehydrated sample reveals the presence of a new phase which contains no traces of crystallites of either γ-CD heptahydrate or pure fisetin (Figure 1). It should be highlighted that the absence of peaks indicating recrystallization of the isolated components is a clear evidence of the formation of a stable and pure inclusion compound [30].

Note also the good similarity between the diffraction envelope of the recrystallized γ-CD·fisetin and the diffractogram calculated from the atomic coordinates of a model inclusion complex, γ-CD–12-crown-4 ether (trace (d) in Figure 1) [31]. Such a match, according to Caira’s systematization of crystalline CD inclusion compounds [30], reflects the isostructurality of the two samples. It is, thus, fair to assume that in the compound γ-CD·fisetin the host–guest units are also stacked in the form of infinite channels. In this kind of structures, the longitudinal axes of the channels are aligned to form squares, as depicted in the inset of Figure 1, a geometry that results in the formation of wide interchannel void spaces which are suited to accommodate a large amount of water molecules. The postulated geometry for the γ-CD·fisetin inclusion compound is thus in agreement with the results of the elemental analysis and of the thermogravimetric analysis (described below).

**Figure 2:**
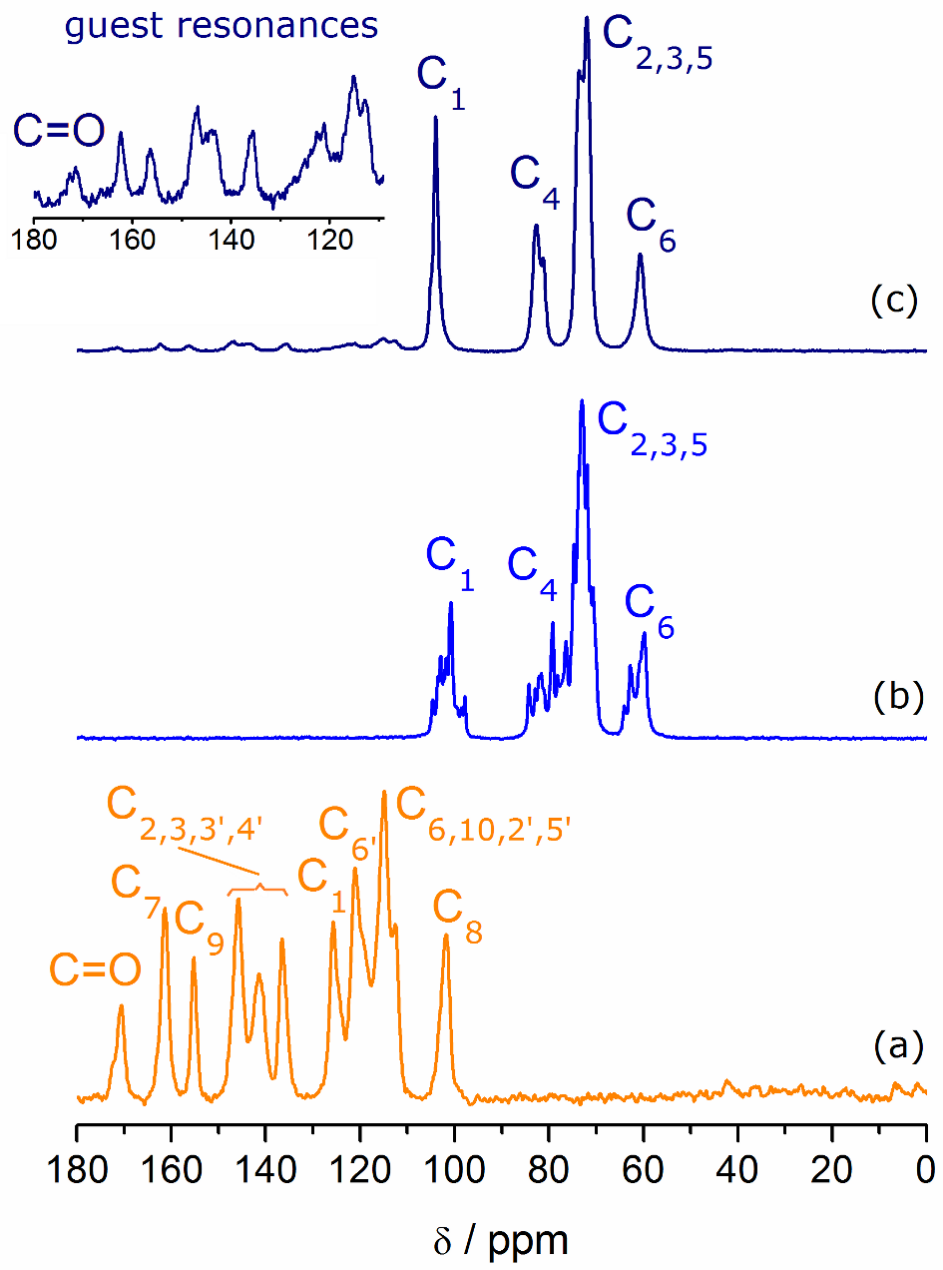
^13^C{^1^H} CP/MAS NMR spectra for (a) fisetin, (b) γ-CD host, and (c) γ-CD·fisetin. The inset shows an expansion of the 180–110 ppm region for the spectrum of γ-CD·fisetin, in which the resonances of the guest are observed.

**^13^C{^1^H} CP-MAS NMR.** The solid-state NMR spectra of fisetin, γ-CD hydrate and γ-CD·fisetin are depicted in Figure 2. The spectrum of fisetin presents some well-resolved resonances that are ascribed to the carbonyl and C1, C7, C8, C9, and C6’ carbon atoms by comparison with its reported solution spectrum [32]. The other resonances are somewhat overlapped and do not allow an individual assignment, in a similar fashion to those observed in the reported solution spectrum [32]. The host, γ-CD, exhibits multiple sharp resonances for each type of carbon atoms. For the C1 and C4 carbons, multiplicity is associated with differences in the conformation about the α(1→4) linkages, while carbons positioned closer to the rims, such as is the case for C6, are more sensitive to ambient changes arising from hydration water molecules and the hydrogen-bonding network [33–34]. Upon inclusion of fisetin, the carbon resonances of the γ-CD loose much of their multiplicity, rather appearing as single signals. Such is the case of C1 and C6, while the signals of C4 and C2, 3, and 5 appear each as a sharp resonance with a shoulder of lower intensity. In agreement with the structural packing in infinite channels, as postulated from the results of PXRD, these single signals for ^13^C further reinforce the symmetrization of geometrical parameters for the γ-CD carbons when they are forming an inclusion compound with fisetin. Regarding the guest signals, the intensities are quite weaker as expected due to the dilution effect caused by the presence of the carbohydrate host, but in an expansion it is possible to discern the presence of almost all carbon resonances of fisetin, with exception of C8, which most likely overlaps with the signal of the C4 of γ-CD. Of note are two resonances that are observed in the region ascribed to the carbonyl of fisetin, indicating the occurrence of at least two different environments or inclusion geometries [35].

**Figure 3:**
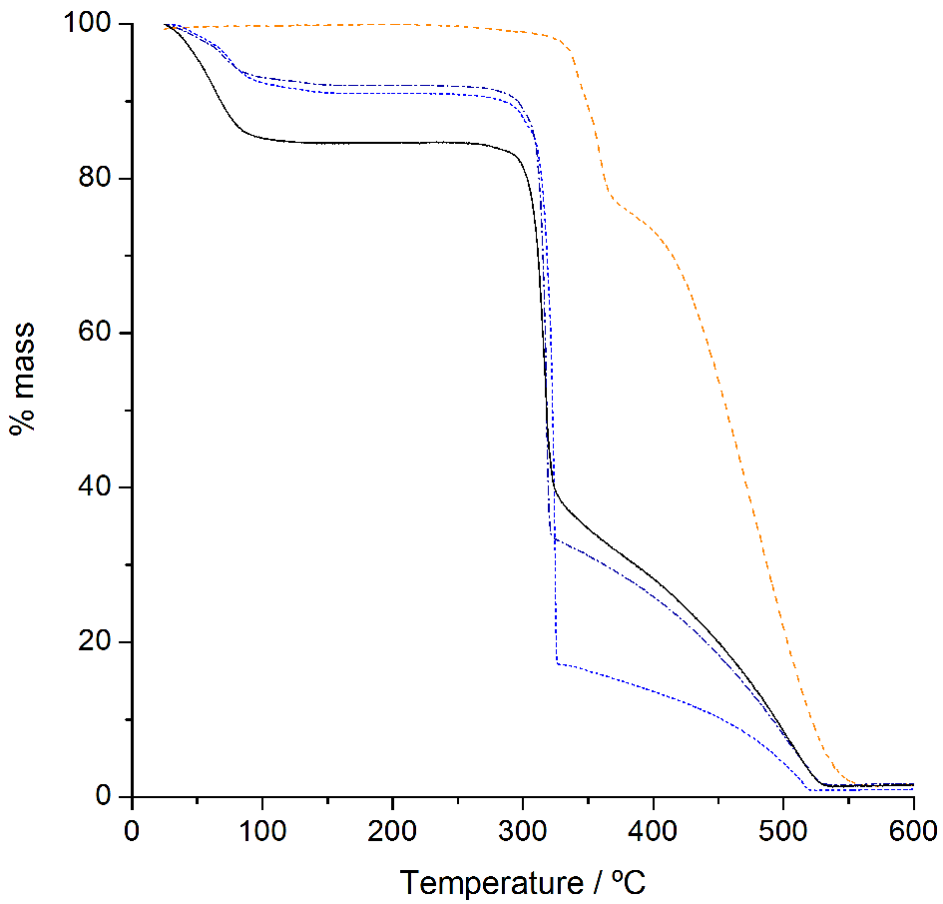
TG traces for fisetin (orange dashed line), γ-CD (blue dotted line), their 1:1 physical mixture (black dashed dotted line), and γ-CD·fisetin (black solid line). The physical mixture was prepared by weighing equimolar amounts of γ-CD and fisetin and gently mixing the two powders with a spatula.

**Thermogravimetric analysis (TG).** In the TG studies, the samples were heated at a rate of 5 °C per min while registering changes in their mass. This allows observing processes of dehydration, sublimation and decomposition [36]. The traces of γ-CD, fisetin, their 1:1 physical mixture and the γ-CD·fisetin inclusion compound are depicted in Figure 3.

As can be seen from the TGA trace for fisetin no mass losses are observed between ambient temperature up to roughly 300 °C, evidencing that this substance is anhydrous (as expected from its high hydrophobicity). In the 310–360 °C temperature interval, there is a mass loss of roughly 20% which can be associated with a reported endothermic melting peak at 346.8 °C [37]. There is then a second mass loss, becoming rather abrupt from 400 °C onwards, and at 560 °C all fisetin is decomposed. The TGA trace for γ-CD presents an initial mass loss of 9%, which is ascribed to the removal of 7 water molecules. No further mass loss is observed until the onset of its thermal decomposition, at ca. 280 °C, after which the charred residue undergoes a slow thermal oxidation from 330 °C until its complete combustion at 520 °C [38]. The thermal decomposition trace of the physical mixture is quite similar to that of γ-CD, having approximately the same number of hydration water molecules. Remarkably, the step associated with the decomposition of fisetin (which should be visible after 310 °C) is missing. Such effect may arise from the thermal protection properties of the cyclodextrin, since these cyclic carbohydrates are known to act as flame retardants [39]. The same effect is also observed in the TG trace of the γ-CD·fisetin inclusion compound, that is, decomposition of the guest is not observed. The most striking difference between the trace of the inclusion compound and that of the physical mixture is observed in the dehydration profile. The mass loss from dehydration in γ-CD·fisetin is around 16%, corresponding to 17 water molecules. The increase in the number of water molecules upon the formation of an inclusion compound is consistent with channel-packed γ-CD molecules having wide interchannel spaces where water can be located, as described in the PXRD subsection.

### Radical scavenging activity

**Figure 4:**
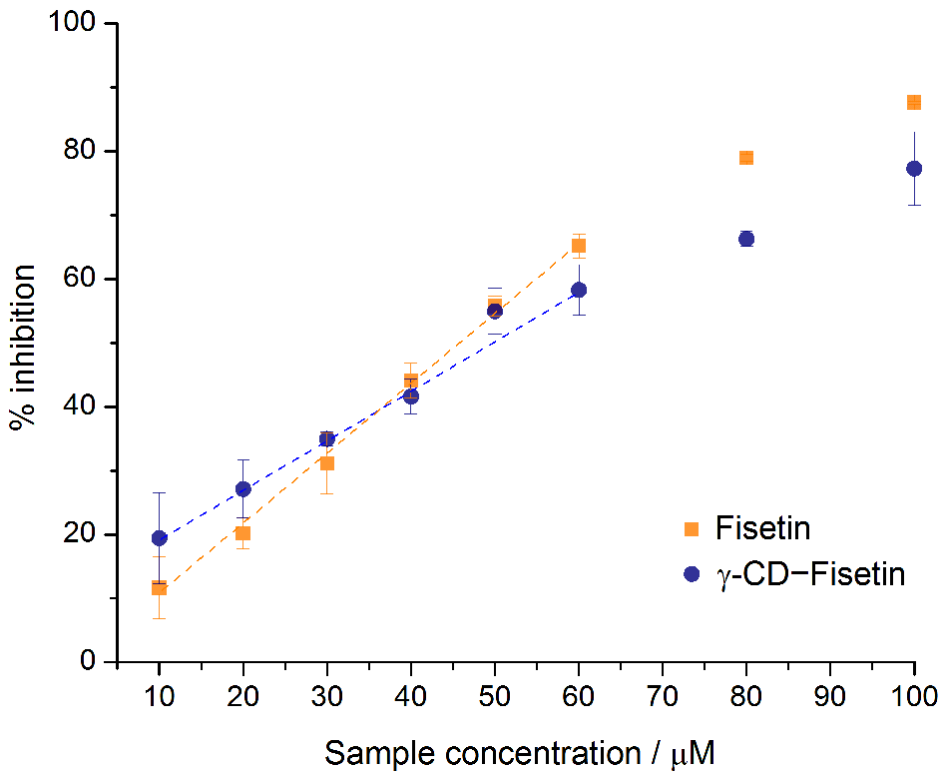
Antioxidant activity of the inclusion compound γ-CD·fisetin, compared with pure fisetin, as determined by the DPPH˙ scavenging assay. Measurements were conducted at the steady-state of inhibition, occurring at 20 min of incubation.

Developed by Blois in 1958 [40], the DPPH assay determines the antioxidant activity of a test compound by using a commercially available stable free radical, DPPH^·^ (2,2-diphenyl-1-picrylhydrazyl) that has a strong violet color. Upon reaction with an antioxidant, DPPH^·^ is converted into the corresponding hydrazine (pale yellow in color), a reaction which can be monitored by UV–vis spectroscopy. As the kinetics of the reaction varies from sample to sample, the stabilization time for a complete reduction of the DPPH^·^ free radical was determined. For all samples studied, the steady-state discoloration time was verified to be 20 min. The percentage of remaining DPPH^·^ in solution was then calculated for each concentration of fisetin and γ-CD·fisetin. From the linear section of the concentration–activity curve for each compound (Figure 4), the effective concentration required to scavenge 50% of the DPPH^·^ (EC_50_) can be determined (Table 2).

**Table 2:** Comparison of the DPPH^·^ scavenging activities of pure fisetin and the γ-CD·fisetin inclusion compound obtained in the present study with the literature results reported for fisetin (at various concentrations of DPPH^·^).

test compound	[DPPH^·^]/μM^a^	solvent	EC_50_/μM	reference

fisetin	14	methanol	14.1 ± 2.1	[41]
fisetin	195	ethanol	54 ± 1.6	[42]
fisetin	250	ethanol	16.0 ± 0.9	[43]
fisetin	152	methanol	49.4 ± 2.2	present study
γ-CD·fisetin	152	methanol	52.6 ± 3.8	present study

^a^Values refer to the final concentration of DPPH in the reaction medium.

Under our experimental conditions, the EC_50_ of fisetin was ca. 50 μM, which remained practically unaltered upon inclusion into γ-cyclodextrin. An attempted comparison with values reported in the literature for fisetin (Table 2) is difficult, as the EC_50_ are quite different. The major reason for this is that the reported studies used different concentrations of DPPH^·^ in the initial reaction mixtures. Note also that a correlation between the different DPPH^·^ concentrations and the corresponding EC_50_ values cannot be established due to insufficient metadata. Further the use of different solvents is also known to affect the results, albeit this influence is associated with changes in the reaction rate and thus it is not expected to cause very significant alterations to the measurements conducted at the steady state.

## Conclusion

In this study, the inclusion complex of fisetin with γ-CD was successfully achieved by co-dissolution followed by co-lyophilization. The material obtained is an amorphous solid powder that can be converted into a microcrystalline powder by rehydration without any dissociation of the inclusion complex. The method can thus be used to preserve fisetin inside molecular capsules for a long time, even in conditions of high humidity.

The solid-sate characterization of the γ-CD·fisetin inclusion compound, particularly by FTIR and ^13^C{^1^H} CP/MAS NMR data, shows that the fisetin molecules are completely included inside the wide cavity of γ-CD, which undergoes a slight geometric rearrangement in order to better accommodate fisetin. This host, the largest of the commercially available native cyclodextrins, is the most compatible with the size of flavonoid compounds. A smaller cyclodextrin, such as β-CD, is not able to fully include them. Having one glucose unit less, the cavity of β-CD is narrower and therefore the tilted structure of a flavonoid does not fit and one of the rings of the guest is positioned outside the cavity. This geometry was recently demonstrated for epigallocatechin, which has the same molecular backbone as fisetin, and features the ring B protruding from the primary rim of β-CD in the crystal structure of the β-CD·epigallocatechin inclusion compound [44]. PXRD of the rehydrated γ-CD·fisetin solid shows that the supramolecular arrangement of γ-CD·fisetin complex units is consistent with channel packing, a structure occurring frequently in solid γ-CD inclusion compounds.

The results from the DPPH scavenging assay show that the antioxidant activity of fisetin is retained upon inclusion, thus making the γ-CD·fisetin inclusion compound a suitable additive for food fortification or health supplements. This inclusion compound combines the antioxidant activity of fisetin with stability and solubility in aqueous media.

## Experimental

### Materials

Fisetin (≥96%) was obtained from TCI Chemicals (Zwijndrecht, Belgium). Pharmaceutical-grade γ-CD (Cavamax W8 Pharma) from Wacker-Chemie was kindly donated by Ashland Specialty Ingredients (Düsseldorf, Germany). Analytical grade ethanol and distilled water were used as solvents.

### Equipment

Elemental analysis was carried out at the Chemistry Department of the University of Aveiro (by M. Marques) on a Leco CHNS TruSpec^®^ Micro elemental analyzer. Laboratory powder XRD data were collected at ambient temperature on an X’Pert MPD Philips diffractometer (Cu Kα_1_ = 1.540598 Å) with a curved graphite monochromator, equipped with a X’Celerator detector, operating in a flat Bragg–Brentano configuration (40 kV, 50 mA). Data was collected with steps of 0.04° in a continuous mode in the 3.5° ≤ 2θ ≤ 50° interval. TG studies were performed on a Shimadzu TGA-50 thermogravimetric analyzer, using a heating rate of 5 °C min^−1^, under air atmosphere, with a flow rate of 20 mL min^−1^. The sample holder was a 5 mm in diameter platinum plate and the sample mass was about 5 mg. Infrared spectra were obtained as KBr pellets in a Mattson 7000 FTIR spectrometer (resolution 2.0 cm^−1^; 64 scans per spectrum). ^13^C{^1^H} CP/MAS NMR spectra were recorded at 100.62 MHz on a (9.4 T) Bruker Avance III 400 spectrometer, with an optimized π/2 pulse for ^1^H of 4.5 μs, 3 ms contact time, a spinning rate of 12 kHz and 4 s recycle delays. Chemical shifts are quoted in parts per million from tetramethylsilane. Absorbance readings for the antioxidant activity assay were measured at the working wavelength of 515 nm in a µQuant™ Microplate spectrophotometer by BioTek Instruments, Inc.

### Methods

**Preparation of γ-CD·fisetin:** To a solution of γ-CD (200.8 mg, 14 mmol) in water (5.0 mL) was added a solution of fisetin (40 mg, 14 mmol) in ethanol (4.5 mL) at 40 °C, and the resulting solution was allowed to stir for 1 h. The mixture was then subjected to snap-freezing, by immersion into liquid nitrogen, and placed overnight in a freeze-dryer to remove the volatiles. A voluminous pale yellow solid was obtained with quantitative yield (240 mg). Rehydration of the solid was achieved by placing it in an open glass container inside a vial containing a thin layer of water at the bottom, thus creating a water-saturated chamber. The solid was allowed to rest inside this chamber overnight (ca. 16 hours). Anal. calcd for C_48_H_80_O_40_·C_15_H_10_O_6_·20H_2_O (1943.7): C, 38.93; H, 6.742; found: C, 38.77; H, 6.481. Thermogravimetric analysis up to 120 °C revealed a mass loss of 16%, corresponding to 17 water molecules. FTIR 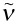 (cm^−1^): 3391 (vs), 2933 (m), 1626 (m), 1609 (m), 1570 (w), 1518 (m), 1463 (m), 1441 (m), 1418 (m), 1372 (m), 1334 (m), 1280 (m), 1254 (m), 1200 (m), 1158 (s), 1123 (sh), 1103 (s), 1079 (vs), 1052 (vs), 1041 (vs), 1029 (vs), 1022 (vs), 1002 (vs), 939 (m), 851 (m), 789 (m), 775 (m), 759 (m), 704 (m), 669 (m), 651 (sh), 608 (m), 580 (m), 530 (m), 480 (m), 462 (m), 446 (m), 413 (m), 358 (m), 325 (w), 315 (w), 308 (w), 296 (w), 290 (w), 281 (w); ^13^C{^1^H} CP/MAS NMR (100 MHz, 12 kHz, 25 °C) δ 170.2 (fisetin, C_4_), 161.2 (fisetin, C_7_), 154.9 (fisetin, C_9_), 145.7, 142.3 (both fisetin, C_2_, C_3’_, C_4’_), 134.9 (fisetin, C_3_), 120.5 (fisetin, C_6’_), 114.2, 111.9 (both fisetin, C_6_, C_10_, C_2’_, C_5’_), 103.9 (γ-CD, C_1_), 82.4, 81.1 (γ-CD, C_4_), 72.5, 71.9 (γ-CD, C_2,3,5_), 60.4 (γ-CD, C_6_). The carbon numbering is given in Scheme 1.

**Spectroscopic data for fisetin:** FTIR and solid-state ^13^C{^1^H} CP/MAS NMR data of pure fisetin were collected and are given below for comparison. NMR attributions were made according to the literature [32]. FTIR 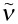 (cm^−1^): 3354 (s), 1620 (vs), 1599 (vs), 1572 (vs), 1510 (vs), 1445 (m), 1421 (m), 1388 (s), 1325 (s), 1279 (vs), 1254 (vs), 1208 (s), 1171 (s), 1134 (m), 1113 (s), 1098 (s), 1015 (m), 978 (m), 935 (m), 883 (m), 846 (sh), 838 (m), 808 (m), 790 (m), 774 (m), 756 (w), 723 (w), 702 (m), 694 (sh), 674 (m), 625 (m), 590 (w), 569 (w), 553 (w), 524 (w), 510 (w), 451 (m), 407 (w); ^13^C{^1^H} CP/MAS NMR (100 MHz, 12 kHz, 25 °C) δ 170.4 (C4), 161.2 (C7), 155.2 (C9), 145.6, 141.2 (C2, C3’, C4’), 136.5 (C3), 125.7 (C1’), 120.9 (C6’), 114.7, 112.4 (C6, C10, C2’, C5’), 101.6 (C8).

**Antioxidant activity by the DPPH method.** The radical-scavenging activity of fisetin and the γ-CD·fisetin inclusion compound were determined using the free radical 2,2-diphenyl-1-picrylhydrazyl (DPPH^·^) [45]. A volume of 25.0 mL of 0.304 mM DPPH^·^ methanol solution was used (the solution was prepared in order to have an abs_510nm_ value in the 0.9–1.0 interval). The reaction was started by mixing 0.1 mL of sample with 0.1 mL of the previously diluted DPPH^·^ methanol solution, in a well of a 96-well plate. Seven dilutions were tested for each sample, with the concentrations of 0.01, 0.02, 0.03, 0.04, 0.05, 0.06, 0.08 and 0.1 mM. The bleaching of DPPH^·^ was followed at 515 nm at 0 min, 5 min, and every 5 min until the reaction reached a steady state. A plateau was reached within 20 min, thus the readings collected at this time were used for calculating the EC_50_ values. The measurements were carried out in three independent experiments.
